# An evaluation of adherence to anti-diabetic medications among type 2 diabetic patients in a Sudanese outpatient clinic

**DOI:** 10.11604/pamj.2019.34.34.15161

**Published:** 2019-09-16

**Authors:** Hyder Osman Mirghani

**Affiliations:** 1 Department of Internal Medicine, Faculty of Medicine, University of Tabuk, Saudi Arabia

**Keywords:** Medications adherence, polypharmacy, HbA1c, diabetes, Sudan

## Abstract

**Introduction:**

Adherence to anti-diabetic medication is a known cornerstone in the management of type 2 diabetic patients. We sought to assess the factors associated with adherence to medication s among type 2 diabetic patients being followed up in a Sudanese outpatient clinic.

**Methods:**

This cross-sectional study conducted among 102 patients with type 2 diabetes attending an outpatient clinic in Omdurman, Sudan during the period from June to December 2017. Participants were interviewed using a structured questionnaire to collect demographic data, number, and type of medications, polypharmacy, medications side effects, financial problems and education regarding drug used. The study of participants’ adherence to anti-diabetic medications was assessed using a validated questionnaire asking the patients about the percent and self-rating of adherence (Excellent, very good, good, fair and poor). The Statistical Package for Social Sciences (SPSS) was used to compare the adherent patients and their counterparts. A P-value < 0.05 was considered significant.

**Results:**

The study results summarized the following: participants (70.6% women), their mean age was (59.62±9.91) years and nearly 60.8% were housewives, their glycated hemoglobin (mean± SD) was about 10.16±3.14, 37.3%, it implies that the patients were non-adherent to medications. In addition, other groups of patients with medication but non-adherence were younger ones (55.94±9.94 vs. 61.81±9.36, P=0.04) and had shown inadequate glycemic control (11.33±3.05vs. 9.47±3.04, P=0.04), however, this group of patients has reported more drug-related side effects (57.8% vs. 28.1%) because they were taking more drugs compared to their counterparts( F=4.115, P=0.047). The present study found no statistically significant differences in the following factors such as sex, occupation, education level, financial problems and insulin use.

**Conclusion:**

In conclusion, the study revealed that adherence to anti-diabetic medications was sub-optimal among Sudanese type 2 diabetic patients and was associated with higher glycated hemoglobin seen among younger age groups. Besides the above, overdosing of medications and their side effects were evident.

## Introduction

Diabetes mellitus is the most common endocrine disease worldwide; it is a serious major health problem. Currently, 285 million were affected, and the projection for the year 2030 is 438 million [1]. According to the World Health Organization estimation, half-million Sudanese people were affected by diabetes mellitus in the year 2000 and this trend is expected to reach one million in the year 2030 [2]. The management of type 2 diabetes includes the adoption of a healthy lifestyle, a friendly diet and pharmacotherapy. Adherence to management plan is an essential component of diabetes holistic care. Adherence is defined as the extent to which a person’s behavior regarding diets, healthy lifestyles, or taking medications or behavior changes coincide with medical and health advice [3]. The lack of adherence to drugs according to doctor prescription has consistently shown to be associated with poor glycemic control and microvascular complications [4, 5] among diabetic patients. Patients’ non-adherence to therapeutic strategies is a common serious health concern worldwide. Medication non-adherence could be related to healthcare system factors, patients’ factors or due to medications. Non-adherent to the prescribed medications is a principal factor in poor glycemic control and microvascular complications including neuropathy, nephropathy and retinopathy with deleterious consequences on the patients and the community as a whole [6]. Polypharmacy, which is the use of simultaneous multiple drugs by the same patient for one or more disease has become a huge problem even in the developed countries [7]. The term polypharmacy, defined as taking five medications or more was shown to increase non-adherence to medications, which lead to a propensity to falls and increase meditational side effects [8, 9]. Previous studies have evaluated the adherence to anti-diabetic medication and polypharmacy in Sudan; however, due to the introduction of new anti-diabetic medications with varying doses and schedules may further affect adherence to medicines behavior, change in lifestyles and culture, which are essential factors to evaluate this critical health problem regularly. Based on the above fact, this survey was designed to assess the adherence to anti-diabetic medications among patients with type 2 diabetes in Sudan.

## Methods

This cross-sectional study conducted at a randomly chosen diabetes clinic from seven clinics in Omdurman, Sudan during the period from June to September 2017. One hundred and two consecutive patients with the diagnosis of type 2 diabetes (according to American Diabetes Association Guidelines) [10] were invited to sign a written informed consent then interviewed using a structured questionnaire to collect age, sex, occupation, their educational level, time since the diagnosis of diabetes, number and type of medications taken by the patients over the period, history of microvascular and macrovascular complications of diabetes if any, financial issues with drugs, and medications side effects, etc. were also included. With the above background, the adherence to anti-diabetic medicines was assessed using the following questions: during the past month what percent of the time did you take all your diabetes medications as your doctor prescribed? And on average, how would you rate your ability to take all your diabetes medications as your doctor prescribed (excellent, very good, good, fair, poor, and very poor)? The short medication adherence questionnaire had been previously validated for use among patients with diabetes [11]. Additionally, the days covered with medication in the past week were reported. On the other hand, non-adherence to anti-diabetic medications was defined as taking less than 80% of the prescribed treatment [12]. A blood sample was taken for the glycated hemoglobin (HbA1c) to assess the degree of glycemic control using the reagent from utilizing a glycol hemoglobin reagent set from HB1C Siemens Healthcare Diagnostics Newark, DE 19714, USA. Patients on five drugs or more were considered as polypharmacy [8]. This study obtained ethical committee approval from the Faculty of Medicine, University of Tabuk to conduct this cohort research. The Statistical Package for Social Sciences (SPSS, Version 20, Chicago) was used for data analysis, the Chi-square test, t-test, and One Way-ANOVA were also used to compare patients who were adherent to medications with their counterparts, a P-value of < 0.05 was considered significant.

## Results

Out of 102 patients with type 2 diabetes (70.6% women), their ages ranged from 28-82 years with a mean of (59.62±9.91 years). Nearly 25.5% were not educated but 29.4% of them had primary education, while 21.6% had a high school education, however, the majority (60.8%) of the patients were housewives and 17.6% were laborer, 15.7% were employed and 5.9% were teachers. Table 1 shows the good glycemic control was evident only in 23.5% of patients. The participants mean HbA1c% was (10.16±3.14), the meantime since the diagnosis of diabetes was (11.90±8.72), the mean number of medications was (4.96±1.35) and the neck circumference was (34.27±9.26cm) (Table 2).

**Table 1:** Basic characters and adherence to anti-diabetic medications among Sudanese patients with type 2 diabetes mellitus

Character	N (%)
**Sex**	
Males	30 (29.4%)
Females	72 (70.6%)
**Education**	
Illiterate	26 (25.5%)
Primary School	30 (29.4%)
Intermediate School	10 (9.8%)
High secondary School	22 (21.6%)
University	14 (13.8%)
**Occupation**	
Housewives	62 (60.8%)
Laborer	18 (17.6%)
Employee	16 (15.7%)
Teachers	6 (5.9%)
Good glycemic control	24 (23.5%)

**Table 2:** Patient's characters mean ±SD

Character	Mean ±SD
Age years	59.62±9.91
HbA1c%	10.16±3.14
Meantime since diabetes diagnosis	11.90±8.72
Number of medications	4.96±1.35
Neck circumference (cm)	34.27±9.26

In the present study, 88.2% were on metformin, 78.4% were taking sulphonylureas, 9.8% were prescribed insulin and 2% were on pioglitazone. It is interesting to note that 92.2% of patients were taking regular vitamins, 35.3% were on regular proton pump inhibitors, 11.8% were on regular non-steroidal anti-inflammatory drugs, while 62.7% were taking five medications or more (polypharmacy). Table 3 illustrates the prescription among patients with type 2 diabetes. The current data showed that 62.7% of patients were adherent to medications, 43.1% rated their adherence as excellent, 31.4% were very good, 21.6% as good, while 3.9% rated themselves as fair. The days covered with medications were seven in 72.5% of patients, six days in 15.7%, five in 2%, 5.9% covered four days, while 2% covered two days. Regarding the negative attitudes of patients regarding medications, sixty-two percent were not adequately educated regarding medication, financial issues were reported by 39.2%, while 37.3% reported medications side effects (Table 4).

**Table 3:** Prescription pattern among patients with type 2 diabetes

Medication	N (%)
Metformin	90 (88.2%)
Sulphonylureas	80 (78.4%)
Pioglitazone	2 (2%)
Insulin	10 (9.8%)
Antihypertensive	58 (56.9%)
Statins	62 (60.8%)
Aspirin (low dose)	56 (54.9%)
Proton pump inhibitors	36 (35.3%)
Vitamins	94 (92.2%)
No steroidal anti-inflammatory drugs	12 (11.8%)
Polypharmacy	64 (62.7%)

**Table 4:** Medication adherence to anti-diabetic medications among patients with type 2 diabetes

N (%)	Character
**Medication adherence %**	
100%	36 (35.3%
90%	26 (25.5%)
85%	4 (3.9%)
80%	12 (11.8%)
75%	8 (7.8%)
70%	8 (7.8%)
60%	2 (2%)
50%	2 (2%)
45%	4 (3.9%
**Adherence self-rating**	
Excellent	44 (43.1%)
Very good	32 (31.4%)
Good	22 (21.6%)
Fair	4 (3.9%)
**Number of days covered with drugs**	
Seven days	74 (72.5%)
Six days	16 (15.7%)
Five days	2 (2%)
Four days	6 (5.9%)
Two days	2 (2%)
One day	2 (2%)
Lack of knowledge about medications	64 (62.7%)
Financial issues	40 (39.2%)
Medications side effects	38 (37.3%)

In the present study, results revealed that patients who were adherent to medications were a mature adult than the non-adherent younger patients (61.81±9.36 vs. 55.94±9.94), P-value=0.040 and these patients had better glycemic control (HbA1c,9.47±3.04 vs. 11.33±3.05) with a significant statistical difference, P-0.040 compared to their counterparts. Besides the above, no significant statistical difference was found regarding the duration of the diagnosis of diabetes (11.93±9.33 vs. 11.84±7.84), P-value=0.970 Table 5.

**Table 5:** The relationship of adherence to anti-diabetic medication, mean age, duration since the diagnosis of diabetes, and HbA1c

Character	Adherence (n=32)	Non-adherence (n=19)	P-value
Age	61.81±9.36	55.94±9.94	0.040
Duration of diabetes	11.93±9.33	11.84±7.84	0.970
HbA1c	9.47±3.04	11.33±3.05	0.040

^*^T-test

The present study showed that adherence was lower among patients who experienced medication side effects (28.1% vs. 57.8%), P-value=0.035, no differences were evident regarding insulin use (6.2% vs. 15.7%), P-value=0.286, sex (31.2% vs. 26.3% of males and 68.8% vs. 73.7% of women), P-value=0.761 and financial issues (37.5% vs. 36.8%), P-value=0.963. Table 6 illustrated the comparison between adherence and non-adherence. In the current study, medication adherence was lower among patients taking higher medications (P-value=0.047, F=4.115, no differences in medications adherence was evident regarding occupation and level of education (P-values=0.483 and0.057, F=0.499 and 3.801 respectively) (Table 7).

**Table 6:** The relationship of adherence to anti-diabetic medication, sex, insulin use and barriers to adherence

Character	Adherence (n=32)	Non-adherence (n=19)	P-value
Sex Males Females	10 (31.2%) 22 (68.8%)	5 (26.3%) 14 (73.7%)	0.761
Insulin use	2 (6.2%)	3(15.7%)	0.286
Financial issues	12 (37.5%)	7 (36.8%)	0.963
Medication side effects	9 (28.1%)	11 (57.8%)	0.035

^*^Chi-square-test

**Table 7:** Relationship of adherence to anti-diabetic medications to the occupation, level of education, and the number of prescribed medications

Character	F	P-value
Level of education	3.801	0.057
Occupation	0.499	0.483
Number of medications	4.115	0.047

^*^One Way-ANOVA

## Discussion

The present study showed that 62.7% of patients with type 2 diabetes were adherent to the prescribed medications with 74% reported their adherence as excellent and very good, and 88.2% had six or more days /week covered with drugs. The present findings were similar to a study conducted in India [6] and reported adherence in 54.6%. Higher rates (74.9%) were reported in Ethiopia [13] and Nepal [14] (97.3%), differences in sample size and the measures used could partially explain the discrepancies between the studies. In the present study, females’ dominance was obvious, the majority were housewives, and more than half received only primary education, a study published in Turkey [15] concluded similar findings.

In the current study, nearly two-thirds of patients were not educated about the medications, 37.3% were concerned about drugs side effects, and 39.2% reported financial issues regarding the medicines to use, previous researchers [6] reported similar results regarding barriers to medications adherence. A recent large study [16] conducted in five European countries observed the agreement regarding metformin as the first-line treatment among patients with type 2 diabetes and differences in the second line medications attributed to different national guidelines. The current data showed the most frequently prescribed medications were metformin followed by sulphonylureas in accordance with Acharya *et al.* [17] who conducted a survey in India and found metformin and glimepiride were the most common medications. The absence of DPP-4 inhibitors and Glucagon-Like Peptide agonists was due to either unavailability or cost.

There is a trend towards increasing combination of statins, aspirin and hypertensive medications among patients with type 2 diabetes [18]. In the present study, more than half of the patients were using these drugs. Previous literature reported that polypharmacy is associated with medication non-adherence, medications side effects, and dementia, in the present study nearly two-thirds of the patients were on five or more drugs in agreement with Patel *et al.* [19] who reported polypharmacy and hyper pharmacy in 59% and 31% respectively. It is interesting to note that 92.2% of the study sample were on regular vitamin supplementation despite the possible harmful effects of regular multivitamins supplementations as fat-soluble vitamins could lead to toxicities due to accumulation in the body. The American Diabetes Association [20] recommended to test serum vitamin B12 and replace if deficient. The present data showed that, 35.3% were on regular proton pump inhibitors in spite of the strong recommendation against their use for more than eight weeks, and their association with Clostridium defficile infections and dementia [21] and 11.9% were taking non-steroidal anti-inflammatory drugs on regular base in spite of the danger of lethal side effects. The pharmacists and the prescribing physicians may need proper communication and counseling of the patients and use of the emerging computer system to optimize the prescription. In the current study, medication non-adherence was higher among patients with poor glycemic control in line with Jemal *et al.* [13] who found an association between non-adherence and poor glycemic control. The relationship between medications side effects and non-adherence to medications is obvious. In the current study, non-adherent patients reported a higher rate of drugs side effects.

The present results found lower medication adherence among patients with polypharmacy in accordance with a previous study [22] which showed a relationship with non-adherence and the complexity of prescription, another study [23] showed adherence to anti-diabetic medication was better among those taking ≥ four drugs than those on lesser drugs and attributed this to the age and level of education in their sample in contradiction to the present findings. In the present study, no differences in adherence were observed regarding sex, occupation, level of education, duration of diabetes, financial problems and insulin use in agreement with Patel *et al.* [20] who concluded similar results. Similar results were observed by Serap and Bayram [15] regarding the association of sex, financial problems, and duration of diabetes with medication adherence, however, the relationship of medications adherence to the level of education was in contradiction to the present findings. In the current survey, non-adherence was common among the young age group and those with higher glycated hemoglobin and in agreement with a population-based study [24] conducted in France, but Tabannick and Fidel [25] recommended that independent variables should not be strongly related to each other. The American Diabetes Association recent recommendations of incorporating agents proven to reduce cardiovascular mortality and major cardiovascular events and the equipoise gave to much second-line therapy may encourage the developing countries to consider the use of new classes of medications in spite of the high cost. Thus, medication class shifting might be a useful method to improve medication non-adherence.

Medication therapy management and better communication of the patients and healthcare providers are highly relevant for therapy optimization and reducing non-adherence and approaching the glycated hemoglobin targets. Further larger multi-center studies to assess adherence to an individual class of medications are highly recommended. The study limitations were the small sample of the research, the reliance on a self-reported questionnaire which could lead to overestimation of the adherence and the fact that the study was conducted at a single diabetes clinic.

## Conclusion

In conclusion, the adherence to anti-diabetic medications was sub-optimal and common among young patients with poor glycemic control, those on five or more drugs and those who exposed to medications side effects, no difference between adherent and non-adherent patients regarding sex, occupation, level of education, the duration since the diagnosis of diabetes, insulin use, and financial problems.

### What is known about this topic

- Polypharmacy is common among patients with type 2 diabetes;
- Inadequate adherence to anti-diabetic medications compromise safety and treatment effectiveness.

### What this study adds

- Non-adherence to anti-diabetic medications was commonmmpm among young type-2 diabetic patients, these patients need more effort regarding the importance of medication adherence;
- Patients who observed medications side effects were non-adherent. Health education regarding antidiabetic side effects may be briefed or needed before the prescription was given. Also, sharing the decision with the patients is vital to enhance medication adherence. Involvement of the pharmacists in the education of patients with type 2 diabetes patients will bring more benefits and of great help to achieve the greater outcome.

## Competing interests

The author declares no competing interests.

## References

[1] International Diabetes Federation Middle East, and North Africa 2015.

[2] World Health Organization Diabetes mellitus: report of a WHO study group.

[3] Ahmad NS, Ramli A, Islahudin F, Paraidathathu T (2013). Medication adherence in patients with type 2 diabetes mellitus treated at primary health clinics in Malaysia. Patient Preference and Adherence.

[4] Khan AR, Al-Abdul Lateef ZN, Al Aithan MA, Bu-Khamseen MA, Al Ibrahim I, Khan SA (2012). Factors contributing to non-compliance among diabetics attending primary health centers in the Al Hasa district of Saudi Arabia. J Family Community Med.

[5] Sankar UV, Lipska K, Mini GK, Sarma PS, Thankappan KR (2015). The adherence to medications in diabetic patients in rural Kerala, India. Asia Pac J Public Health.

[6] Divya S, Nadig P (2015). Factors contributing to non-adherence to medication among type 2 diabetes mellitus in patients attending tertiary care hospital in south India. Asian J Pharm Clin Res.

[7] Sherman JJ, Davis L, Daniels K (2017). Addressing the polypharmacy conundrum. US Pharmacist.

[8] Fried TR, O'Leary J, Towle V, Goldstein MK, Trentalange M, Martin DK (2014). Health outcomes associated with polypharmacy in community-dwelling older adults: a systematic review. Journal of the American Geriatrics Society.

[9] Pasina L, Brucato AL, Falcone C, Cucchi E, Bresciani A, Sottocorno M (2014). Medication non-adherence among elderly patients newly discharged and receiving polypharmacy. Drugs Aging.

[10] American Diabetes Association (2010). Summary of revisions for the 2010 clinical practice recommendations. Diabetes Care.

[11] Gonzalez JS, Schneider HE, Wexler DJ, Psaros C, Delahanty LM, Cagliero E (2013). Validity of medication adherence self-reports in adults with type 2 diabetes. Diabetes Care.

[12] Delamater AM (2006). Improving patient adherence. Clinical Diabetes.

[13] Jemal A, Abdela J, Sisay M (2017). Adherence to Oral Antidiabetic Medications among Type 2 Diabetic (T2DM) patients in chronic ambulatory wards of Hiwot Fana specialized University Hospital, Harar, Eastern Ethiopia: a cross sectional study. J Diabetes Metab.

[14] Sapkota S, Brien JE, Aslani P (2018). Nepalese patients' anti-diabetic medication taking behaviour: an exploratory study. Ethn Health.

[15] Serap T, Bayram S (2015). Factors influencing adherence to diabetes medication in Turkey. Sch J App Med Sci.

[16] Overbeek JA, Heintjes EM, Prieto-Alhambra D, Blin P, Lassalle R, Hall GC (2017). Type 2 diabetes mellitus treatment patterns across Europe: a population-based multi-database study. Clin Ther.

[17] Acharya KG, Shah KN, Solanki ND, Rana DA (2013). Evaluation of antidiabetic prescriptions, cost and adherence to treatment guidelines: a prospective, cross-sectional study at a tertiary care teaching hospital. Journal of Basic and Clinical Pharmacy.

[18] Lafeber M, Grobbee DE, Spiering W, van der Graaf Y, Bots ML, Visseren FL, SMART Study Group (2013). The combined use of aspirin, a statin, and blood pressure-lowering agents (polypill components) in clinical practice in patients with vascular diseases or type 2 diabetes mellitus. Eur J Prev Cardiol.

[19] Patel PJ, Hayward KL, Rudra R, Horsfall LU, Hossain F, Williams S (2017). Multimorbidity and polypharmacy in diabetic patients with NAFLD: Implications for disease severity and management. Medicine (Baltimore).

[20] American Diabetes Association (2018). Summary of revisions: standards of medical care in diabetes-2018. Diabetes Care.

[21] Mere SE, Paauw DS (2017). Common drug side effects and drug-drug interactions in elderly adults in primary care. J Am Geriatr Soc.

[22] Jackson Idongesit L, Adibe Maxwell O, Okonta Matthew J, Ukwe Chinwe V (2015). Medication adherence in type 2 diabetes patients in Nigeria. Diabetes Technology & Therapeutics.

[23] Adisa R, Fakeye TO (2013). Effect of number and type of antidiabetes medications on adherence and glycemia of ambulatory type 2 diabetes patients in southwestern Nigeria. Pharmacy Practice.

[24] Tabannick BG, Fidel LS (2008). Using multivariate statistics.

[25] Tiv M, Viel JF, Mauny F, Eschwège E, Weill A, Fournier C (2012). Medication adherence in type 2 diabetes: the ENTRED study 2007, a French Population-Based Study. PLoS One.

